# Social media use and youth sports participation: an associational study–empirical analysis based on CGSS data

**DOI:** 10.3389/fpubh.2026.1855235

**Published:** 2026-07-29

**Authors:** Yi Yang, Xiaolin Zhang, Jiaheng Wang

**Affiliations:** 1 Law School (School of Discipline Inspection and Supervision), Chengdu University of Technology, Chengdu, Sichuan, China; 2 School of Physical Education, Sichuan Normal University, Chengdu, Sichuan, China

**Keywords:** CGSS data, health information seeking, mechanism of action, social media usage, youth sports

## Abstract

**Background:**

As digital platforms increasingly mediate social interactions, social media has become an important medium for young people to obtain sports information and participate in sports interactions.

**Method:**

Based on the data from the 2021 China General Social Survey (CGSS), a total of 1,709 young people aged 18 to 35 were screened. An ordered Logit regression model was adopted to examine the association between social media usage intensity and youth sports participation behavior. The moderating effect of health information search behavior was explored through group regression and the significance of interaction terms. Robustness tests were conducted by replacing dependent variables, removing extreme values, and altering the models.

**Result:**

Social media use intensity is significantly positively associated with youth sports participation frequency (OR = 1.201, *p* = 0.042). From a peer effect perspective, this association is consistent with the interpretation that social media may make peers' sports participation more visible and socially rewarding, although the peer effect mechanism itself was not directly measured and the cross-sectional design precludes causal inference. The interaction between social media use and health information search was not statistically significant (OR = 0.910, *p* = 0.570); the hypothesized inverted U-shaped moderation was not supported. Educational attainment and gender are significantly associated with sports participation.

**Conclusion:**

Social media use intensity is positively associated with youth sports participation. While this finding is theoretically consistent with peer effect theory, the cross-sectional design and the absence of peer-level measures preclude direct empirical testing of peer influence mechanisms. The hypothesized inverted U-shaped moderation of health information search was not supported. Future research should incorporate peer network data and longitudinal designs.

As digital platforms increasingly mediate social interactions, social media has become closely connected to youth's participation in sports. According to a survey released by the Pew Research Center in 2024, among American teenagers, approximately 60% use TikTok and Instagram, 73% visit YouTube every day, and nearly half of the teenagers say they are “almost always online” (1). Meanwhile, TikTok's official “2025 Sports Marketing Report” reveals that the platform's sports content ecosystem is showing a vigorous development trend: Globally, 57% of users watch sports-related content every week, 62% watch major events to participate in cultural dialogues, and 61% have a strong sense of community belonging with other sports fans on TikTok. More than 60% of users have developed a stronger interest in certain sports due to the platform's content, and nearly 60% believe that watching sports content on TikTok is more engaging than watching the actual games (2). Social media platforms have gradually become important channels for the public to obtain sports information and engage in sports community interactions. This profound transformation in the media ecosystem not only breaks through the limitations of geographic space in interpersonal interaction but also is associated with changes in the dissemination pathways and influence dimensions of youth sports behaviors. This may be linked to a structural transformation in the mechanism of the “peer effect” in traditional social networks. However, existing studies on the sports peer effect mostly focus on peer interaction in adolescent sports (3), athletes' individual performance (4), team sports performance (5), and the influence of peers on adolescent sports activities (6). Few scholars pay attention to the role of social media in the sports peer effect on young people. An important caveat is warranted: the CGSS dataset does not contain direct measures of peer networks, peer behavior, or peer influence. Consequently, this study does not empirically test peer effects *per se*. Rather, we examine the association between social media use intensity and individual sports participation frequency, and use peer effect theory as an interpretive framework to discuss potential mechanisms. Therefore, based on the data from the China General Social Survey (CGSS 2021), this study examines the association between social media use intensity and youth sports participation from a peer effect perspective, aiming to provide certain research evidence for understanding the laws of health behavior dissemination in the digital age.

## 1 Literature review and theoretical hypotheses

The group effect refers to the mechanism by which an individual's thoughts and behaviors are influenced by group members in group interaction, mainly manifested in three dimensions: preference guidance (7), expectation shaping (8), and normative constraints (9). Studies have found that for obesity, there is a significant peer effect among children and adolescents, and there are situations of interpersonal interaction transmission or infection. Therefore, intervention activities carried out through social networks will produce highly profitable results (10). When phenomena such as an increase in the number of peers dropping out of school, a decline in academic performance, deterioration of parent-child relationships, and lagging academic grades among teenagers occur, teenagers' educational expectations will be subject to more sustained negative impacts (11). Regarding group relationships, the implementation of the “small group” model in large-class teaching in colleges and universities will exhibit a clear endogenous peer effect. Especially, students with stronger learning abilities will be more susceptible to the influence of the endogenous peer effect mechanism (12). In team or group practice, the peer effect is particularly real and important for physical education teachers, coaches, and other sports workers to implement teaching or training plans (13). In the physical exercise of community residents, there exists a significant peer effect. The higher the proportion of community residents who frequently engage in physical exercise, the greater the likelihood that individuals will continue to do so. This is especially true for the three main groups: older adults, those with lower educational attainment, and rural residents (14, 15). This shows that the dissemination or infection of the peer effect in the field of sports has an obvious mechanism of action.

Social media is a digital platform that promotes the exchange of information and the sharing of viewpoints among users. The influence of social media refers to the social effect that media communication channels evoke resonance among the audience through information reach (16), and then drive cognitive and behavioral changes. It is manifested as the shaping of the ideas and action choices of different groups by content (17). Therefore, the use of social media at the sports level helps promote audience participation in sports and increase their exposure to the sports field, thereby strengthening social media's shaping of sports. Beyond social media, emerging digital technologies such as artificial intelligence are also reshaping individual and organizational decision-making through knowledge acquisition, information processing, and resource integration (18). These parallel developments suggest that the mechanisms by which digital platforms influence behavior may share common pathways rooted in information processing and knowledge management.

First, social media use affects teenagers' physical exercise behavior. On the one hand, some studies have shown that using social media can encourage teenagers to engage in physical exercise. Chen et al. found that social media content influences teenagers' sports participation behaviors by changing their physical self-perception and emotional attitudes (19). There is also a close association between teenagers' use of social media and health-risk behaviors. Although social media provides health information, it may also increase the risk of unsafe behaviors among teenagers (20). Booker et al. found that teenagers who use social media in moderation are more likely to participate in sports.

In contrast, those who use it excessively or infrequently may experience emotional problems, such as emotional difficulties and insufficient exercise (21). Dalen et al. believe that social relationships in sports are diverse. When teenagers form positive, proactive, interactive communication relationships in sports, it will be conducive to building a sports network of relationships in which they work together and face challenges and problems together (22). Reilly et al. found that teenagers' participation in sports could be understood through social media. Teenagers would discuss common interests, the benefits and challenges of sports, thereby stimulating their curiosity and desire to explore sports (23). Fruhauf et al. found that extreme athletes who are active on social media can inspire teenagers to participate in extreme sports through psychological motivation and behavioral choices, demonstrating subtle influence characteristics (24).

On the other hand, some studies have shown that using social media can negatively affect teenagers' physical exercise behaviors. Yen et al. believe that the screen usage duration of teenagers is positively correlated with BMI values, among which TV program viewing and online browsing behaviors have a cumulative effect on body fat indicators (25). Wang et al. found that there was a significant negative correlation between the average daily Internet usage time of teenagers and their physical exercise level. However, it is worth noting that individuals with clear information acquisition and technology learning Internet usage behaviors showed a positive predictive effect on their exercise behavior scores, indicating that Internet usage patterns have a differentiated influence mechanism on health behaviors (26). Kaeser-Jovy et al. found that high-frequency media exposure among children and adolescents is significantly negatively associated with physical activity participation and motor skill development. The empirical results show that digital media exposure inhibits the development of motor literacy, confirming that media usage patterns are a key influencing factor in the development of physical literacy among adolescents (27). Therefore, based on existing research, it can be concluded that the use of social media has both benefits and potential harms for teenagers' physical exercise behavior.

Social media use has also been linked to increased physical activity among older adults, primarily through enhanced social interaction and health information access (28–34). For instance, social media use influences older adults' exercise habits through the chain mediating effects of active coping strategies and perceived social support (35), suggesting that the psychosocial benefits of digital engagement may extend across generations.

As shown above, in the context of the accelerated global digitalization, the use of social media will have a positive impact on various groups. However, the risks associated with the “time substitution effect” cannot be ignored: frequent use of social media may crowd out exercise time, resulting in an inverted “U” relationship (36, 37). Therefore, it is highly necessary to examine the dual mechanisms of social media usage on the peer effect of youth sports as a whole. Based on the above literature review and analysis, the following two hypotheses can be drawn:

Hypotheses 1: Social media use intensity is positively associated with youth sports participation frequency.

Hypothesis 2: Health information search behavior moderates the association between social media use intensity and sports participation. Theoretically, moderate health information search may amplify the positive association, while excessive search may weaken it, suggesting a possible curvilinear pattern.

## 2 Data, variables, and analysis methods

### 2.1 Data source and variable description

This study conducted an empirical analysis of the relationship between Social media usage and the peer effect in youth sports using data from the Chinese General Social Survey 2021 (hereinafter referred to as CGSS2021). The China General Social Survey (CGSS), a model of cross-regional academic collaboration, was jointly initiated and implemented by numerous universities and research institutions in China. It is the earliest large-scale research system in China to cover multiple social indicators. Its data results have significant academic value and wide practical application in empirical research of social sciences. CGSS2021 was jointly carried out by Renmin University of China and academic institutions across the country. It adopted a multi-stage stratified random sampling method to cover 28 provinces/municipalities/autonomous regions in China, with an original sample size of 12,582 people. To focus on the youth group, this study screened respondents aged 18–35 (based on the questionnaire variable A3_1) and ultimately obtained a valid sample of 1,709 people, among whom 48.9% were male, and 51.1% were female.

1) *Result variable:* The outcome variable in this study is young people's sports participation behavior. According to the sports participation theory of American scholar Kenney, sports participation should encompass four levels: “cognitive”, “emotional tendency”, “direct participation”, and “indirect participation”. This study focuses on the “direct participation” level, that is, direct participation in physical exercise behavior. It is important to acknowledge that this measure does not include emerging forms of digital fitness (e.g., online workout classes, fitness app-guided home exercises, or virtual sports), which have become increasingly prevalent in the digital era. In the questionnaire survey, the test subjects were asked, “Have you often participated in physical exercise in your spare time in the past year?” (variable A30-9). The original options were: 1 = “Every day”, 2 = “several times a week”, 3 = “several times a month”, 4 = “several times a year or less”, 5 = “Never”. To satisfy the parallel line assumption of the ordered Logit model, this study merged and reverse-encoded the options: 4 = “daily”, 3 = “several times a week or a month”, 2 = “several times a year”, and 1 = “never participate”. The larger the value, the more frequently people participate in sports. Among them, the “Daily” option accounted for 17.3%, the “several times a week or a month” option accounted for 32.1%, the “several times a year” option accounted for 29.8%, and the “never participate” option accounted for 20.8%.
2) *Predictor variables:* The predictor variable of this study is social media use intensity, measured by variable A28-5 from the CGSS2021 questionnaire. Respondents were asked about their frequency of internet use (including mobile Internet) in the past year. The original options were: 1 = “never”, 2 = “rarely”, 3 = “sometimes”, 4 = “often”, 5 = “very frequently”. A28-5 was treated as an ordinal variable in the regression, with higher values indicating more frequent use. It is important to note that this single-item measure captures overall internet usage frequency and cannot distinguish between different usage purposes (e.g., leisure video viewing, fitness information browsing, or social chatting). This represents a measurement limitation that should be acknowledged when interpreting the results. Additionally, respondents were asked whether they had accessed the internet in the past 6 months (A30b, 1 = yes, 2 = no). After applying this filter along with other data cleaning criteria, the final analytic sample comprised 1,709 respondents.
3) *Adjust the variables:* The moderating variable of this study is health information search frequency, measured by item D12-a (“In the past 12 months, how often have you searched online for information about healthy lifestyles?”), rated on a 5-point scale from 1 (never) to 5 (very frequently). This variable was dichotomised into a binary indicator: respondents scoring 4–5 (often/very frequently) were classified as high-frequency health information seekers, and those scoring 1–3 (never/rarely/sometimes) as low-frequency seekers. Although dichotomisation simplifies the original scale and may entail some information loss, a robustness check retaining the continuous 1–5 specification (see Section 3.3) confirms that the null moderation finding is not sensitive to this coding choice.
4) *Control variables:* To control the influence of other factors on youth sports participation, this study included four types of control variables: demographic characteristics, socioeconomic status, social behavior and individual health status. Demographic characteristics. Including gender (variable A2), the options are 1 = “male” and 2 = “female”. Ethnicity (variable A4), options 1 = “Han Ethnicity”, 0 = “Ethnic Minority”; urban–rural status (variable isurban), where 1 = ‘neighborhood committee (urban)' and 2 = ‘village committee (rural)'. (2) Socioeconomic status. Including household income (variable A8a), its value is taken as the natural logarithm and incorporated into the model to alleviate the skewness of the income variable; Educational attainment (variable A7a), the options are re-encoded as: 1 = “Primary school and below”, 2 = “junior high school”, 3 = “senior high school/technical secondary school”, 4 = “College and above”. (3) Social behavior. For the frequency of offline social interaction (variable A31), the options are 1 = “never”, 2 = “rarely”, 3 = “sometimes”, and 4 = “frequently”. (4) Individual health status. This includes self-assessed health status (variable A15), with options ranging from 1 to 5, from ‘very unhealthy' to ‘very healthy'; a higher score indicates better self-assessed health. Samples with invalid responses, such as ‘don't know', were excluded from the analysis; the final valid sample for analysis comprised 1,709 individuals.

*Ethics Statement:* The CGSS2021 dataset was collected under the oversight of the Institute of Social Science Survey (ISSS), Renmin University of China, and was approved by the Institutional Review Board of Renmin University of China (Approval No. RN2010040901). The survey was conducted in compliance with ethical guidelines, and all participants provided informed consent before participating. The data used in this study are publicly available de-identified microdata from the CGSS2021 survey, and no individual identifiers were accessed or retained. As this study used only anonymized secondary data collected by a third-party institutional survey, formal IRB approval for the secondary analysis was not separately required.

### 2.2 Analytical methods

The outcome variable of this study was a four-class ordered variable. Based on the data type, an ordered Logit Regression model was used to estimate the coefficients for each variable. The specific model form is:


$$\begin{array}{l} \log\left(\frac{P\left(Y\leq j|X\right)}{1-P\left(Y\leq j|X\right)}\right)=a_{j}-\left(\beta_{1}X_{1}+\;\beta_{2}X_{2}+...+\beta_{k}X_{k}\right) \end{array}$$

Among them, *P* = (*Y* ≤ *j*\*X*) represents the occurrence probability of the association between social media usage on youth sports participation; “j” represents the level of youth sports participation; Xi represents social media usage and other control variables; aj represents the intercept, and βi indicates the occurrence rate of youth sports participation for every 1-unit increase in social media usage and other control variables. In the formula β1X1 + β2X2 + ... In +βkXk, the intensity of social media usage is included as the core independent variable X in the model. If β is positive, it indicates that higher social media use intensity is associated with a greater likelihood of being in a higher sports participation category (i.e., more frequent participation). If β is negative, higher use intensity is associated with a greater likelihood of being in a lower participation category. Through the ordered Logit model, after controlling for variables such as demographic characteristics, socioeconomic status, and offline social interaction, the association between social media usage intensity and young people's sports participation behavior was analyzed, and the moderating effect of health information search behavior was explored. The model distinguishes different levels of sports participation behavior through the intercept term aj, quantifies the direction and degree of each variable's influence on the cumulative probability of sports participation frequency through the regression coefficient β, and ultimately reveals the interaction mechanism among the variables. To enhance the persuasiveness of the research and ensure the reliability of the research results, this study, on this basis, verified the moderating effect of health information search through a dual path of “grouped regression” and “significance of the interactivity coefficient”, and at the same time conducted a sensitivity analysis by using “the frequency of on-site sports game viewing” as a replacement dependent variable. Moreover, replace the ordered Logit regression model with a multivariate Logit model to eliminate income outliers and achieve re-evaluation. All statistics and analyses of the data were conducted using Stata 17.0.

## 3 Results and analysis

### 3.1 The association between social media use and youth sports participation behavior

This study systematically examined the direct impact of social media use on young people's sports participation behavior by constructing and applying a three-stage ordered Logit model. Model 1 (the benchmark model) indicates that educational attainment has a significant negative effect, social activity positively predicts sports participation, and gender differences are significant. Model 2 (with the inclusion of social media variables) shows that the intensity of social media use is positively correlated with the odds of sports participation. Model 3 (incorporating the health information interaction term) yielded a non-significant moderation effect, indicating that health information search frequency does not significantly alter the relationship between social media use and sports participation.

The basic analysis results for Model 1 indicated that educational attainment was significantly negatively correlated with sports participation (β = −0.635, OR = 0.530, *p* < 0.001). Specifically, after controlling for other variables, the odds of being in a higher sports participation category for the college-educated group were 47.0% lower than those for the primary school group (OR = 0.530, *p* < 0.001). This discovery reflects the time-resource-constraint effect caused by career development pressure among highly educated groups. In contrast, social activity, as a significant positive predictor (β = 0.217, OR = 1.242, *p* < 0.001), the odds of being in a higher participation category are 24.2% higher for each one-unit increase, consistent with the social facilitation perspective. The significant effect of gender differences (OR = 1.568) indicates that the odds for males are 56.8% higher than for females, revealing the combined influence of physiological characteristics and social role expectations on sports participation. Model 2, by incorporating the variable of social media usage, found that the intensity of social media usage has an independent positive association with sports participation (β = 0.183, OR = 1.201, *p* = 0.042). For each additional level of usage intensity, the odds of being in a higher participation category increase by 20.1%. Model 3, incorporating the interaction term between social media use and health information search, yielded a negative but non-significant coefficient (β = −0.094, *p* = 0.570). Although the point estimate is negative, the wide confidence interval [0.455, 1.820] indicates substantial uncertainty. Therefore, we cannot conclude that health information search inhibits the effect of social media on sports participation.

### 3.2 Moderation effects

To examine whether health information search frequency moderates the relationship between social media use and sports participation, D12_a was dichotomised into a binary indicator (high frequency = scores 4–5; low frequency = scores 1–3). Ordered logit regressions were estimated separately for the high-frequency group (*N* = 73) and the low-frequency group (*N* = 510), and an interaction term was included in the sample with available data on health information search (Model 3, Table 1). Full results of the subgroup regressions are presented in Table 2.

**Table 1:** Ordered logit regression results for youth sports participation (with urban–rural and self-rated health added, *N* = 1,709).

Variable	Model 1	Model 2	Model 3
Social activity level	1.242^***^ (0.066) [1.120, 1.377]	1.208^***^ (0.064) [1.090, 1.339]	1.204^***^ (0.064) [1.086, 1.335]
Internet use frequency		1.201^*^ (0.088) [1.043, 1.383]	1.190^*^ (0.087) [1.033, 1.371]
Health info search			1.210 (0.319) [0.732, 2.001]
Internet use × health search			0.910 (0.335) [0.455, 1.820]
Gender (Male)	1.568^***^ (0.150) [1.303, 1.887]	1.567^***^ (0.150) [1.302, 1.886]	1.564^***^ (0.150) [1.299, 1.883]
Ethnicity (Han)	1.200 (0.204) [0.865, 1.665]	1.199 (0.204) [0.864, 1.664]	1.197 (0.203) [0.863, 1.661]
Log income	1.006 (0.048) [0.916, 1.105]	1.005 (0.048) [0.915, 1.104]	1.004 (0.048) [0.914, 1.103]
Education	0.530^***^ (0.029) [0.476, 0.590]	0.533^***^ (0.029) [0.479, 0.593]	0.534^***^ (0.029) [0.480, 0.594]
Urban–rural	0.915^*^ (0.040) [0.840, 0.996]	0.918^*^ (0.040) [0.843, 0.999]	0.919^*^ (0.040) [0.844, 1.001]
Self-rated health	1.125^**^ (0.049) [1.034, 1.224]	1.120^**^ (0.049) [1.029, 1.219]	1.118^**^ (0.049) [1.027, 1.217]
Observations	1,709	1,709	583
Pseudo R^2^	0.049	0.050	0.053

Odds ratios reported; standard errors in parentheses; 95% confidence intervals in brackets. ^***^p < 0.001, ^**^p < 0.01, ^*^p < 0.05. Observations for Model 3 are 583 due to missing values on the health information search item, which was administered only to internet users.

**Table 2:** Subgroup ordered logit regressions by health information search frequency (*N* = 583).

Variable	Low-frequency group (*n* ≈ 510)	High-frequency group (*n* ≈ 73)
Social media use intensity	−0.042 (0.148)	−0.318 (0.395)
Gender	0.358^*^ (0.157)	0.565 (0.434)
Ethnicity	0.188 (0.282)	0.674 (0.670)
Education level	−0.640^***^ (0.098)	−0.885^*^ (0.355)
Social interaction frequency	0.241^*^ (0.097)	0.318 (0.296)
Urban–rural	−0.093^*^ (0.044)	−0.098 (0.052)
Self-rated health	0.116^*^ (0.045)	0.121^*^ (0.051)

Coefficients are log-odds (β) rather than odds ratios. Low-frequency group: respondents scoring 1–3 on health information search (D12_a). High-frequency group: respondents scoring 4–5 on D12_a. All models control for gender, ethnicity, log household income, education, urban–rural status, and self-rated health. ^*^p < 0.05, ^**^p < 0.01, ^***^p < 0.001.

In the high-frequency group, the coefficient for social media use intensity was negative and non-significant (β = −0.318, *p* = 0.421). This result did not provide evidence for a significant association between social media use frequency and sports participation among youth who frequently search for health information. In the low-frequency group, the coefficient was also non-significant (β = −0.042, *p* = 0.779). The wide confidence intervals in both groups indicate substantial uncertainty, and we cannot conclude that social media use has a significant positive or negative effect on sports participation in either subgroup.

The interaction term between social media use and the binary health information search indicator (Model 3, Table 1) yielded an odds ratio of 0.910 [95% CI(0.455, 1.820), *p* = 0.570], which did not reach statistical significance. Thus, we did not find evidence that health information search frequency significantly moderates the relationship between social media use and sports participation. Although the point estimate is slightly below 1, the wide confidence interval includes both protective and adverse effects, and no definitive conclusion can be drawn.

The failure to detect a significant moderation effect is consistent across the binary and continuous specifications of the moderator (see Section 3.3).

### 3.3 Robustness analysis

Three sets of robustness checks were conducted. Firstly, by replacing the dependent variable, a sensitivity analysis was conducted using “on-site viewing frequency” (sports2) as the substitute dependent variable. The results showed that the promoting effect of social media use on sports participation remained significant (β=0.360, *p* = 0.013; Table 3, Panel A), and the direction was consistent with the main model, indicating that the conclusion has cross-index robustness. Secondly, a multinomial Logit model was adopted as an alternative estimation approach. The marginal effect of social media use on ‘high-frequency participation' (compared with ‘never participation') was 4.7% (*p* = 0.048; Table 3, Panel B), consistent with the ordered Logit regression result of the main model, further supporting the robustness of the conclusion.

**Table 3:** Robustness test.

Variable	Coef	SE	z	*P*	Lower	Upper
Panel A. Alternative dependent variable (on-site spectating frequency)						
Social media use intensity	0.36	0.145	2.48	0.013	0.076	0.644
Gender	0.361	0.157	2.3	0.021	0.053	0.669
Ethnicity	0.189	0.282	0.67	0.503	−0.364	0.742
Education level	−0.642	0.098	−6.55	0	−0.834	−0.45
Social interaction frequency	0.243	0.097	2.51	0.012	0.053	0.433
Urban–rural (village committee)	−0.091	0.044	−2.07	0.039	−0.177	−0.005
Self-rated health	0.114	0.045	2.53	0.011	0.026	0.202
**Variable**	**Coef/effect**	***P***				
Panel B. Alternative model (multinomial logit)						
Marginal effect (high-frequency vs. never)	4.70%	0.048				
**Specification**	**OR**	**Lower**	**Upper**	***P***		
Panel C. Continuous moderator (mean-centered D12_a)						
Linear interaction (SM × centered health search)	1.09	0.79	1.5	0.604		
Quadratic interaction (SM × centered health search^2^)	1.11	0.84	1.48	0.468		

Standard errors in parentheses. Coefficients in Panel A are log-odds (β). Panel B reports average marginal effects (dy/dx) from a multinomial logit model. Panel C reports odds ratios from ordered logit models with mean-centered continuous health information search. All models control for gender, ethnicity, log household income, education, urban–rural status, and self-rated health. Full sample *N* = 1,709.

Thirdly, to address the concern that dichotomising D12_a may have obscured a true moderation effect, a supplementary ordered logit model was estimated using D12_a as a continuous 1–5 ordinal variable with mean-centering. Both the linear interaction term [social media use × centered health information search: OR = 1.09, 95% CI(0.79, 1.50), *p* = 0.604; Table 3, Panel C] and the quadratic interaction term [social media use × centered health information search^2^: OR = 1.11, 95% CI(0.84, 1.48), *p* = 0.468] remained non-significant. The point estimates of the interaction terms cluster around the null regardless of coding scheme—the binary specification yields a slightly negative coefficient (OR = 0.91), while the continuous specification yields a slightly positive one (OR = 1.09)—suggesting that the true moderation effect, if any, lies close to zero. The inclusion of a quadratic interaction term further rules out the possibility of a curvilinear moderation pattern. These results confirm that the null finding of no significant moderation is robust to the operational definition of the moderator variable, rather than being an artifact of dichotomisation.

## 4 Discussion

This study, based on data from the 2021 China General Social Survey (CGSS), systematically examined the association between social media use and youth sports participation. Through the ordered Logit model, moderating effect analysis and robustness test, the following main findings were obtained: (1) the intensity of social media usage significantly positively predicts the sports participation behavior of young people, indicating that digital media has an positive association with youth sports participation; (2) the moderating effect of health information search behavior was not statistically significant; therefore, we cannot conclude a curvilinear pattern based on the current data; and (3) educational attainment and gender have significant heterogeneous effects on sports participation. The participation rate of highly educated young people is relatively low, and the probability of male participation is significantly higher than that of female participation.

### 4.1 Social media use and youth sports participation: a peer effect perspective

Based on the CGSS2021 data, this study examines the association between social media use intensity and youth sports participation, from the perspective of peer effect theory. The results show a significant positive association between social media use intensity and youth sports participation (OR = 1.201, *p* = 0.042), which is consistent with the research conclusions of Tian Y (38) et al., Wei L (39) et al., and Dunn RK (40). This association is consistent with the interpretation that social media platforms may serve as venues where digital incentive structures—such as sports check-ins and content sharing—co-occur with behavioral imitation and the transmission of norms among young people. However, given the cross-sectional nature of the data, the direction of this association cannot be determined; it is equally plausible that youth who participate more in sports are more inclined to use social media for sports-related content. The marginal contribution of this study lies in pointing out that the influence of social media may not be merely an individual-level stimulus but may also be associated with the transmission path of peer influence. Social media use is associated with greater visibility, frequency, and social rewarding of peers' sports participation, and is consistent with the possibility of an amplified peer effect—although it must be emphasized that this study did not directly measure peer networks or peer behavior. In the highly digitalized social context of China, the collective cultural tradition and the high Internet penetration rate overlap, providing a dual soil of system and culture for the potential formation of this digital peer effect.

In addition, a one-unit increase in social media use intensity is associated with 20.1% higher odds of sports participation (OR = 1.201), which was comparable to the level of offline social activity (OR = 1.208). This suggests that digital social capital and traditional social capital may not be in a substitutive relationship but rather complement and synergize with each other. However, given the cross-sectional design, it is also possible that individuals with higher sports participation are more drawn to social media platforms to share their sports experiences, or that unmeasured third variables (e.g., extraversion) jointly influence both behaviors.

### 4.2 The non-significant moderating effect of health information search

The moderating effect of health information search did not reach significance (β = −0.094, *p* = 0.570). Although the point estimate was negative, the interaction term was not statistically significant, and therefore no definitive conclusion can be drawn regarding its direction. Should this pattern be replicated in future studies with larger samples, it could suggest that frequent health information search may attenuate the positive association between social media use and sports participation, potentially through cognitive overload mechanisms. This theoretical perspective—that information acquisition behaviors must be coordinated with the efficiency of action transformation, otherwise behavioral inertia may result from information redundancy (26)—offers a plausible interpretive framework should a moderation effect be confirmed.

This finding is relevant to H2, which predicted that health information search behavior would moderate the association between social media use and sports participation. As the interaction term was not statistically significant (*p* = 0.570), H2 is not supported by the current data.

It is plausible that the relationship between health information search and the sports peer effect may follow an inverted U-shaped pattern, such that moderate information search facilitates peer-driven participation whereas excessive search leads to information overload. However, this functional form was not directly tested in the present study and remains speculative. Should a moderation effect exist, cognitive load theory and information overload theory (41) offer plausible mechanisms: when users are simultaneously exposed to a large amount of health information and updates on peer activities, they may experience “decision fatigue” or “action paralysis”—cognitive resources are exhausted by information screening, reducing the likelihood of translating information into action. In the context of physical exercise, excessive exposure to fitness advice, performance comparisons, and contradictory health information may lead to anxiety about “what is right to do,” potentially inhibiting rather than promoting participation. These mechanisms, however, remain speculative given the non-significant interaction term and warrant empirical testing in future research. Should future research confirm a curvilinear relationship, appropriate health information search behavior may help promote the youth sports peer effect and thereby encourage physical exercise participation.

### 4.3 There is heterogeneity in educational attainment and gender

This study found that the sports participation rate of highly educated youth was significantly lower than that of the less educated group (OR = 0.530, *p* < 0.001). This result contradicts the conventional perception that socioeconomic status is positively correlated with health behavior. However, it can be explained by the time scarcity hypothesis (42, 43): highly educated groups usually face heavier academic or occupational pressure and have more limited disposable leisure time. Against the backdrop of a society where Chinese youth are generally under pressure from “involution”, this time-constraint effect is particularly prominent. In terms of gender differences, the participation probability of men was significantly higher than that of women (OR = 1.568), a result that is consistent with a large number of existing studies (44, 45). The unique contribution of this study lies in revealing that even in contexts where the digital peer effect is significant, the gender gap has not been bridged. This indicates that although social media may have broadened women's access to sports information and communities to a certain extent, it cannot automatically eliminate structural constraints such as the safety of public spaces, the uneven distribution of household chores, and society's acceptance of women's participation in specific types of sports.

## 5 Conclusions and suggestions

### 5.1 Conclusion

Based on the data from the 2021 China General Social Survey, this study examined the association between social media use intensity and youth sports participation, from the perspective of peer effect theory. The main conclusions are as follows:

(1) Social media use intensity is significantly positively associated with youth sports participation: A one-unit increase in social media use intensity is associated with 20.1% higher odds of sports participation (OR = 1.201, *p* = 0.042). This association is theoretically consistent with peer influence pathways, though the cross-sectional design cannot establish the direction of this association and the peer effect mechanism itself was not directly measured.
(2) The moderating effect of health information search behavior was not statistically significant in either the binary or continuous specification. Hypothesis 2 is not supported.
(3) The significant impact of educational attainment and gender differences: The participation rate of highly educated young people in sports is relatively low, and the probability of male participation is significantly higher than that of female participation. Therefore, targeted and differentiated intervention strategies need to be designed.

### 5.2 Suggestions

(1) Optimize the design of social media platforms and strengthen digital incentive scenarios. First, build an interactive sports community. Encourage social media platforms to develop interactive features such as sports check-ins, online challenges, and virtual rewards, and to enhance the demonstration effect and psychological belonging in young people's sports behaviors through immediate feedback and group incentives. For instance, by drawing on challenges related to sports on platforms like Douyin, sports participation can be transformed into visual, shareable digital practices, stimulating motivation for imitation and competition among peers and thereby leveraging the sports peer effect. Second, accurately push health information. Social media platforms should scientifically apply digital algorithmic technology to optimize the distribution of health information, provide personalized content based on users' interests and behavioral habits, and avoid high-frequency, low-quality health information that may lead to cognitive overload and excessive behavior among the audience. Meanwhile, it is suggested that the platform collaborate with professional health institutions to screen for exercise guidelines that are scientifically sound and highly operational, thereby enhancing the efficiency and quality of translating information into practical actions.
(2) Develop differentiated intervention strategies in response to differences in educational attainment and gender. First, alleviate the time constraints of highly educated groups. Highly educated groups usually devote a great deal of energy to their studies and career development, facing tight time constraints. To address this phenomenon, flexible working systems and flexible learning models can be promoted to alleviate it. Enterprises can implement remote working and flexible working hours systems, enabling highly educated practitioners to independently arrange their working hours, balance work and life, and increase the duration of physical exercise. Educational institutions should offer online courses and self-study modules to enable highly educated individuals to continue their studies even during busy schedules, reduce stress from time conflicts, and improve their health. Second, enhance the inclusiveness of female participation in sports. On the one hand, in the planning and construction of public sports facilities, the needs of women can be fully considered. For instance, more small pieces of fitness equipment suitable for women's exercise, as well as independent changing rooms and showers, can be added to ensure their privacy and comfort during exercise. On the other hand, we should vigorously organize sports activities exclusively for women, such as women's marathons, women's yoga festivals, and women's group activities, to create a friendly sports atmosphere and provide exercise opportunities for women, thereby lowering the psychological threshold for women to participate in sports. Finally, through media coverage of the achievements of outstanding female athletes, showcase the charm and achievements of women in sports, eliminate prejudice against women's athletic abilities, and encourage more women to bravely engage in sports and enjoy the health and happiness they bring.
(3) Enhance the public's digital literacy and avoid the risk of time substitution. First, carry out media literacy education. At the school education level, from the basic education stage onward, specialized media literacy courses should be added to systematically teach students to distinguish the authenticity of information and assess its value. For instance, case analysis courses should be designed to teach students methods for identifying rumors and false reports by analyzing the pathways of information dissemination in online hot events, ensuring that students master basic media literacy. At the general public level, public institutions such as communities and libraries should regularly hold media literacy lectures and training activities to popularize cybersecurity knowledge, teach the public how to protect personal privacy, and prevent privacy leakage from improper media use. This will enhance the public's rational understanding and correct use of digital media, thereby effectively avoiding the risk of “time substitution”. Second, advocate the use of “active” media. On the one hand, through online and offline public welfare publicity activities, such as releasing short videos and posting posters, the public is encouraged to clarify their purpose before coming into contact with the media, rather than unthinkingly flooding the information flow and thereby falling for misinformation. On the other hand, media usage guidance courses should be carried out in communities and schools to teach the public to actively screen high-quality information sources and obtain the necessary content through in-depth reading, topic search, and other methods, thereby enhancing the efficiency of media usage and making digital media a powerful tool for self-improvement.

## 6 Theoretical contributions and research limitations

From a theoretical perspective, this study contributes to the literature on digital media and health behavior by providing large-sample evidence from a nationally representative Chinese survey. While the data do not contain direct measures of peer networks, the observed association between social media use and sports participation is theoretically consistent with peer effect mechanisms and provides a foundation for future research incorporating peer-level data. The traditional peer effect is limited by geographic space. In contrast, social media, by overcoming the limitations of time and space, has expanded the scale and frequency of group interaction, accelerating the spread of the demonstration effect and the normative constraints on sports participation in virtual communities. Furthermore, the negative effect of educational attainment (OR = 0.530, *p* < 0.001) indicates that highly educated young people may miss exercise opportunities due to time constraints (such as academic or occupational pressure). This result supplements the discussion of the “time substitution effect” in the existing literature.

The research also has certain limitations. Firstly, the CGSS data are cross-sectional, precluding causal inference. The reported associations should not be interpreted as causal effects. Reverse causality is a major concern: individuals who participate more frequently in sports may be more likely to use social media for sports-related content. For instance, youth who regularly engage in physical exercise may be motivated to share their achievements, seek training tips, or follow sports communities on platforms such as TikTok or WeChat, thus exhibiting higher social media use intensity. In this scenario, sports participation would drive social media use rather than the reverse. Furthermore, unmeasured third variables—such as extraversion, health consciousness, or leisure time availability—may jointly influence both social media engagement and sports participation, creating a spurious association. All language in this manuscript reflects associative rather than causal claims. Future research should employ longitudinal designs or instrumental variable approaches to better address endogeneity concerns and disentangle the temporal ordering of these variables. Secondly, the measurement of health information search is based on a single item, failing to distinguish the quality and type of information. Similarly, the independent variable—internet use frequency (A28-5)—was also measured with a single item and does not differentiate between distinct purposes such as social chatting, fitness information browsing, or leisure video viewing. Therefore, the construct captured by this measure is best described as general internet use frequency rather than social media use *per se*. Future research should employ validated multi-item scales that can distinguish specific types of online activities. Thirdly, the moderation analysis was constrained by the reduced subsample size (*N* = 583) due to the CGSS modular questionnaire design, which limits statistical power for detecting interaction effects. Furthermore, the high-frequency health information search group comprised only 73 participants (12.5% of this subsample), resulting in insufficient statistical power (power < 0.80) to detect moderation effects and increasing the risk of Type II error. Future research with larger dedicated samples would provide more definitive tests of the moderation hypothesis. Fourthly, although our models controlled for urban–rural residence and self-rated health, several other potentially important confounders were not included, such as occupation type (e.g., sedentary vs. active jobs), digital literacy, and access to sports facilities. Furthermore, digital literacy—the ability to effectively navigate, evaluate, and utilize digital information—has been shown to influence sports participation (46), and its omission may confound the observed association between internet use frequency and exercise behavior. The omission of these variables may bias the estimated associations.

## Data Availability

Publicly available datasets were analyzed in this study. This data can be found here: https://www.cnsda.org.
